# Abstracts and Gleanings

**Published:** 1884-02-20

**Authors:** 


### ABSTRACTS AND GLEANINGS.

**European vs. American Surgeons.**—Dr. Eugene Smith, Professor of Ophthalmology and Otology in the Detroit Medical College, who is now for the third or fourth time "doing" the great European centres, sends us the following notes from London :

"Taking it for granted that some of your readers may be interested in a short letter from this country, and that it may carry an occasional one back to old times and old stamping ground, I concluded to take a little time from my own pleasure and devote it to the readers of the Medical Age. Having visited this country several times for study and pleasure, I flatter myself that I am competent to compare notes.

"I do not hesitate to 'give the broom' to American Surgeons, regarding them, of course, as a class. A world of surgery is done at the London hospitals, and, as a rule, rougher surgery can hardly be imagined, but that it is successful I do not doubt. Very naturally, I visit principally the eye and ear hospitals and departments of other hospitals devoted to the specialty, but, so far I have failed to find such operators as Bowman, Chritchett and Soelberg Wells, (the first retired and the two latter dead). At Moorfield nearly all the old corps of ophthalmic surgeons are gone.

"I saw one surgeon make the most peculiar and bungling operation for cataract I ever witnessed. He made an incision in the upper part of the cornea with a Beer's cataract knife, finishing the cut, after the puncture, by a come and go, see-saw movement, without a counter-puncture. Of course there was a deal of dragging, and after his iridectomy was made I was not surprised to see the lens pass upward under the sclera instead of presenting in the wound and coming out as it should. It was extracted with a spoon and a quanity of vitreous escaped. It is very doubtful if the old lady will see.

"Operations in other branches of surgery were equally rough. As an illustration I could cite an excision of the knee, performed by two surgeons of Guy's Hospital, last week.

"That delicate consideration for the poor sufferers which prevails with Americans seems either blunted or entirely wanting on this side of the big pond.

"I would advise young men recently graduated to get several years' practice at home before visiting this country. When, from experience, they are able to sift the wheat from the chaff, there is no city in the world that will compare with London in its advantages. The clinics are so arranged a person can visit one hospital in the morning and another in the afternoon, and employ his time in this way the entire week. At each hospital he will find several surgeons and physicians at work, and can choose such as he will visit. I consider the London elinics superior to those of the continent.

"A visit to the new Hotel Dieu, the Hospital de la Charite and other Paris hospitals, affords much of interest to any physician, whether or not he is conversant with the French language. I, of courae, have been most interested in visiting the clinics I attended years ago. De Wecker has, perhaps, the largest, and is probably the grertest experimenter, while De Meyer is more conservative and has the good of his patients more at heart. Galezowski has a large clinic and is doing an immensity of good work. In this country experiments are so general that I do not wonder that so much that is new emenates from it, but I am satisfied that the patient's welfare is better studied in America than here. I have had the pleasure of introducing a new treatment for episcleritis to the oculists over here, and as it has never been mentioned in print, perhaps it would be well to say a word concerning it now. The disease, fortunately rare, is one of the most troublesome to treat. During the past year and a half we have treated at St. Mary's Hospital Eye Infirmary five or six cases successfully with a solution of eserine (sulphate), two grains to the ounce, once or twice a day, dropping two drops in the conjunctival sac. I first used it to counteract unpleasant symptoms arising from the use of atropia, and the result was so surprising that we have used it ever since in these cases. Several severe cases have been cured in from 8 to 10 days, whereas by the old methods with atropia and potas. iod., weeks or months were required.

"I saw at De Wecker's clinic some of the cases of granular lids treated by jequirity, and the Doctor was kind enough to give me a quantity of the seeds to try the treatment at home. I am skeptical, and, in fact, the Doctor does not think it will cure all cases; but it seems to have worked wonders in some patients. I shall give it a fair treal and hope that it will prove successful, for trachoma is a great bug-bear in Michigan."—*Med. Age.*

**Medicine by Inunction.**—Dr. J. C. Amis (Louisville Medical News) reports the following case treated by inunction:

B., white, female, aged two years six months, was attacked, August 17th, with remittent fever, which rapidly developed symptoms of great gravity. Treatment began with quinia by the mouth; but, owing to the extreme irritability of the stomach, little or none of the medicine was retained. The case grew worse from day to day until, on August 23d, I considered it hopeless, there being present well-marked cerebral symptoms. The temperature was 104°; the stomach so irritable that it would not retain even a spoonful of mint-water. The features were pinched, and the skin was livid and covered with clammy perspiration. I gave one-drop dose of the tincture of aconite root, which lowered the temperature one degree and the pulse six beats. I then ordered sixty grains of quinia in one ounce of lard, and the child was anointed with this every three hours for two days. When I returned on the second day, I found that the case had made a decided change for the better. The symptoms of brain trouble had disappeared, and the child was no longer restless, but had had a refreshing sleep. The temperature was 102°, and there was some nausea. I now determined to give this plan of treatment a full trial. A warm bath was ordered to remove the oil from the skin and to facilitate absorption ; and the inunction was repeated every four hours.

When I next saw this patient, three days later, convalescence was well established. A tonic of iron was now prescribed. The patient's recovery was rapid and complete.

Since this time I have treated several other cases of malarial fever by this method and with perfectly satisfactory results. Besides carrying the medicine into the system, I believe that the skin absorbs a good part of the oil also, which. if true, is a most important feature of the treatment, since it enables the physician to exhibit in the same prescription supportive and specific remedies at times when both are urgently demanded, and when, from the condition of the alimentary canal, they could not be otherwise successfully administered.

**Collodion.**—Mr. Sampson Gamgee, one of England's ablest surgeons, writes, in the Birmingham Medical Review :

"To swollen parts which cannot well be bandaged, collodion is especially applicable for the compression attending its contraction. I was lately consulted in the case of a good-looking boy considerably disfigured by a red and swollen nose, which became very pale and visibly contracted just after I painted it with successive layers of collodion. I repeated the application three times in the succeeding fortnight, with the effect of producing shrinkage of the organ to its normal size and color.

"When the nasal bones are fractured, a very effective mode for keeping them immovable, after adjusting them with the fingers, may be thus made : place over the nose a thin layer of absorbent cotton soaked in collodion ; as it dries another layer of cotton and more collodion, taking care that the application extends sufficiently on each side to give a buttress-like support. The patient compares the feeling to the application of a firm bandage on the nose, and the bones consolidate effectively under the shield, which may be renewed as it cracks and peels off.—*Louisville Med. News.*

THE Therapeutic Gazette, for December, contains a symposiac on cascara sagrada, from which we select some facts which do not seem to have been very generally familiar. Dr. C. W. Tangeman, of the Medical College of Ohio, has subjected it to a series of physiological experiments, the results of which he contributes as follows :

1st.—Cascara sagrada, when given in small doses (fifteen to twenty drops), acts like a vegetable bitter on the stomach ; it increases the flow of gastric juice, stimulates the peptic glands to increased action, thereby bringing about healthy gastric digestion.

2nd.—It acts on the sympathetic nervous system, sending an increased blood supply to the intestines.

3rd.—It increases to a limited extent peristaltic action of the small bowels, but increases it very much in the colon, and especially in the rectum.

4th.—It has a specific action on the rectum in the way of peristalsis, to cause this portion of the bowel to unload itself.

5th.—It does not affect the passage of the food in the small intestines any more than a bitter tonic would.

6th.—It is not a safe remedy in pregnancy or uterine disorders, especially when given in cathartic doses.

7th.—It does not affect the larger glandular organs, liver, pancreas or spleen, even when given in cathartic doses.

8th.—Hypodermically the remedy will never produce the permanent good results in chronic constipation that are obtained when it is given by the mouth.

9th.—When employed subcutaneously it acts simply as an evacuant to the rectum.

10th.—The same quantity given hypodermically that produces marked effects when administered by the mouth, will not have the same effect clinically or physiologically.

Dr. T. L. Wright, of Bellefontaine, Ohio, discusses the peculiar applicability of cascara cordial, of which rhamnus purshiana is the base, in the treatment of the constipation of elderly persons. In this class of cases many of the symptoms which are usually associated by physical decay are directly traceable to constipation, and Dr. Wright has found that cascara cordial, through its tonic-laxative properties, removes this condition greatly to the improvement of the person's spirits.—*Ex.*

**The Treatment of Delirium Tremens.**—In spite of all that has been written on this subject, Dr. Atkinson (Practitioner) thinks that sufficient attention is not given to the necessity of supplying the system with adequate nourishment. Certainly in the second stage, when the appetite is bad, the blood-corpuscles deficient in quantity and shrivelled, and the brain anæmic and starved, it is useless to expect relief from sedatives unless the brain is at the same time supplied with the nourishment it requires. Death, no doubt, in delirium tremens, arises from want of sleep, but then it must be remembered that the want of sleep is caused by want of nourishment. The most important part of the treatment, then, is to improve the quality of the blood as quickly as possible by throwing into the system frequent supplies of light, nourishing and easily digestible food. The best way of accomplishing this end is by cutting off all stimulants and ordering half a tin of Brand's liquid essence of beef and half a pint of milk, to be taken alternately every two hours. As regards medicine, twenty-five grains of chloral with thirty minims of compound tincture of cardamons in an ounce of water, taken every four hours, after the beef tea, will be found most useful. Very little effect, though, is produced by the first dose of chloral, inasmuch as the brain is without the nourishment it requires, but after the second dose of the food begins to tell—some sleep, generally speaking, results, and this goes on increasing in proportion as the support is maintained. If nourishment is withheld, sleep disappears, and the old delirium returns.

By this treatment the patient is generally free from all delusions in about twenty-six hours, but good, strong liquid food must still be taken for some days, though not quite so frequently. When there have been from ten to twelve hours more or less continuous sleep, then it is advisable to give up the chloral and give thirty minims of the compound tincture of gentian with five minims of the tincture of nux vomica three times a day for about three days. This restores the tone of the nervous system and stomach and creates an appetite. A little tincture of euonymin may next be substituted for the nux vomica, and some Carlsbad salt may be given in the morning when required. By this treatment the duration of the delirium and the after-effects of the alcohol are very much lessened.—*Medical Index.*

**Treatment of Fractures by Plaster of Paris Dressing.**— The following bit of practical information is taken from a Bellevue Hospital lecture, reported by Dr J. H. Woodward for the Medical News:

Plaster of paris is most frequently and most successfully used here in the form of the plaster of paris bandage. The bandage should be made of crinoline or cheese cloth. Crinoline will hold more plaster in its meshes, and on that account it is the better material to use. Crinoline (or cheese cloth) is cut into strips two and a half or three inches wide, and eight or twelve yards long. These strips are rolled up moderately tight, like the ordinary roller bandage, and while they are being rolled a thin, even layer of dry plaster of paris, recently baked, is spread upon the upper surface of the crinoline. The bandage, being thus thoroughly filled with the plaster, is wrapped in a piece of newspaper and placed in a covered tin box, where it can be kept dry until used.

Splints are constructed with this bandage in the manner to be described immediately. For example, simple fractures of the tibia and fibula are often put up within a few hours after admission to the hospital. If the surgeon thinks there will be any unusual swelling of the limb, he does not hesitate to apply the immovable splint. In such cases a very thick layer of ordinary cotton is wrapped about the foot and leg, from the toes to just below the knee. An assistant maintains the bones in good position while the splint is being made. The paper is now removed from the plaster bandage, and it is placed on end in luke warm water, which is just deep enough to cover the bandage, a handful of salt having been added to the basinful of water. The bandage is squeezed two or three times to facilitate the entrance of the water into it, and when saturated (*i. e.*, after the lapse of about a minute) it is removed from the water and squeezed as dry as possible. About one foot of it is then unrolled and cut off; the remainder is passed snugly over the layer of cotton, by the figure-of-eight turn, from the toes to just below the knee. The bandage applied thus should be smoothed with the hands, and dry plaster of paris may be rubbed into it. A second and a third layer are put on in the same way. Three layers of this bandage make a very firm splint. Two layers are quite sufficient for the first splint, if you propose to keep the patient in bed during the first ten days after the receipt of injury. If the limb is considerably swollen, the patient is usually kept in bed until the first splint is loose. Under such circumstances two layers of bandage are sufficient.

Having enveloped the fractured limb in two (or three) layers of the plaster of paris bandage, it is placed upon a soft pillow which is moulded extemporaneously, so that the position of the splint will not be flattened or the leg bow backwards. Sand-bags, or some substitute, should be placed beside the leg to keep it quiet and prevent angular deformity, and the foot should be kept flexed upon the leg, either by a sand-bag or some other support. The patient is cautioned to keep very still. This splint, exposed to the air, will harden satisfactorily in from one to three hours.

Unless signs of strangulation of the leg appear, and they must be looked for diligently when this immovable apparatus is applied so early in the case, or the patient complains of unusual pain in the part, the splint may remain upon the leg until it is loose, *i. e.*, for a week or two. It is then cut down with a strong, sharp knife. Before cutting, nitric acid may be applied along the line of incision to soften the plaster. Having removed the splint and the cotton, you should examine the limb and correct any deformity. The fractured bones should be maintained in good position by an assistant while the second, and usually final, splint is applied. A roller bandage of old blanket, or of thick flannel, should be passed around the limb in a spiral, covering from the toes to just below the knee. Then three layers of the plaster of paris bandage are applied by the method already described. The bandage should be laid upon the limb smoothly and snugly. If it is put on lightly, the splint will be loose as soon as the plaster is hard; if it is applied tightly the veins may be strangulated. Wind the bandage on snugly, and the splint will be a success. This splint should be treated, while the plaster is setting, just as described for the first splint. As soon as the plaster is hard the patient may go about on crutches.—*Peoria Med. Monthly.*

**The True Nature of a "Cold."**—Dr. C. E. Page writes as follows, touching the nature of a "cold," in the Popular Science Monthly for January: Years of study and observation have forced me to the conclusion that the disease which manifests the symptoms popularly supposed to indicate that a cold has been caught, is to all intents and purposes a filth-disease, arises largely from indigestion, and forms the basis, so to say, or is in fact the first stage of all the so-called filth-diseases. Whatever interferes with digestion or depuration, or depraves the vital organism in any manner, produces an impure condition of the body—a condition of disease; and a continuance of disease-producing habits must inevitably result in periodical or occasional "eruptions," the severity of which will depend upon the degree of one's transgression. Among the causes of this impure bodily condition are (1) impure food, (2) excess in diet, and (3) impure air. Our homes, offices, shops, halls, court-houses, churches, and, with rare exceptions, all living-rooms, private or public, are insufficiently or not at all ventilated; and, except while in the open air, a very large portion of our people, in all the walks of life, habitually breathe an atmosphere vitiated by being breathed over and over again; they are starving for want of oxygen, and are being poisoned by carbonic acid. In default of sufficient oxygen the best of food cannot be transformed into pure blood—there will always be a corresponding indigestion; nor can the carbonic acid be eliminated freely in an impure atmosphere. We have, then, serious "interference with digestion and depuration" whenever we remain even for a single hour of the twenty-four in an "in-door" atmosphere, *i. e.*, an atmosphere that is not in tolerably free communication with the great body of air without. The only offset for restriction in oxygen is restriction in diet and exercise; but a combination of this character would produce enfeeblement of the system, though if a proper balance were maintained there would arise no febrile symptoms such as we are considering. We have plenty of people living in unventilated rooms who, as far as *exercise* is concerned, live a well-balanced life; but seldom do these, any more than the robust and active, practice any sort of voluntary restriction as to quality or quantity of food—nausea and lack of appetite being the only safeguards. Persons of this class are great sufferers from colds.—*Med. Age.*

**Antiseptics in the Puerpera.**—We have for some time past given our preference almost exclusively to Listerine as an antiseptic in the puerpera. We think it has real advantages over any other, while it certainly is much more acceptable to the patient. The objects to be attained in the use of vaginal washes after confinement are, first, to secure cleanliness; second, to arrest and prevent putrefaction; and last, but by no means least, to promote early, healthy action in the lacerated soft part—channels through which septic matter may be absorbed. The importance of this early reparative action cannot be overestimated, as it is a well-known fact that with the healing process once begun, septic virus is repelled, in lieu of being absorbed, as is the case before healing begins or when the abraded surfaces have taken on unhealthy action. We had long employed the carbolic acid for the purposes above enumerated, but observing disagreeable constitutional influences from its use, we selected the Listerine, which has proven a powerful non-toxic antiseptic, promptly promoting the reparative process, and substituting an agreeable perfume for a noxious odor.—*Prof. F. L. Sims, M.D., Memphis College Hospital, in Mississippi Valley Monthly.*

**On the Action of Agaricin in the Nightsweats of Phthisis.**—The agaricus albus, of now almost obsolete reference, as growing upon the larch in the old country, and considered as an active purgative, has been recently brought into use from the fact that the chemists have extracted the active principle, in the form of long needle-shaped crystals, to which the name has been given of Agaricin. Andrel found the Agaric in powder in doses of eight grains, gradually increased to a drachm, useful in the nightsweats of phthisis. And now Dr. Otto Seifert calls our attention in the *Weiner Medizinische Wochenschrift* to the agaricin as used for the same purpose. It is not positive in its action and must often be given in increasing doses, but it induces sleep, rclieves cough and lowers the pulse. According to Dr. Seifert's experience, a full dose exerts its influence during five to six hours, when, knowing the period for the onset of the sweats in phthisis, it must be renewed for that purpose. He gives the drug in doses of 0.004 to 0.02 gramme; and has met with success by using it hypodermically, his formula being: Agaricin, 0.05; alcohol abs., 4.5; glycerine, 5.5; producing a pretty severe burning sensation for half an hobr. He gives preference to the internal administration.—*Jour. Amer. Med. Association.*

**Treatment of Exophthalmic Goitre.**—From an experience of upward of seventy cases, and fortified by the unanimous observations of Von Dusch, Eulenburg, Meyer, Erb and others, Dr. Chvostek is led to regard the rational employment of galvanism as the most important part of the treatment of Basedow's disease. He recommends the following method to be pursued: (1) the ascending constant current applied to the cervical sympathetic, on each side, for at the most one minute; (2) the same to the spinal cord (the anode at about the fifth dorsal spine, the cathode high up in the cervical region); (3) through the occiput (one pole at each mastoid process), and in certain cases also through the temples, a constant current, for at the longest one minute, and so weak that the patient can feel but the slightest sensation of burning. Sometimes, also, local galvanization of the thyroid gland with a weak constant current for about four minutes, the current to be reversed at the end of each minute. The applications should be made every day if possible. As a rule very good results were obtained, even in the most severe cases a cure or marked improvement being recorded. In three cases death resulted from excessive anemia or complications.—*Centralbl. fur klin. Med.; Practitioner.*

**Vesication in Diphtheria.**—Dr. W. F. Bartlett, of Buffalo, New York, communicates to the Therapeutic Gazette for December the results of his experience in the use of cantharidal blisters in diphtheria. His plan is to apply the blister immediately on the appearance of the exudate in the throat. The theory is that the materies morbi is eliminated through the blistered surface, while the counter-irritation thus caused relieves, also, the engorged pharyngeal surfaces. He regards the exudate in the throat as merely an announcement of the presence of the poison in the blood, and that from the nature of the epithelium or impinging of inspired air primarily upon those surfaces, the partial elimination of the morbid element is accomplished.—*Ex.*

**Corrosive Sublimate in Gonorrhœa.**—Dr. Joseph McChesney, of Deming, New Mexico, contributes to the Therapeutic Gazette, for December, a report of a series of seven cases of gonorrhœa in which he employed, by way of treatment, only a solution of corrosive sublimate, one grain to six ounces of water. The results are already very surprising. In several of these cases this injection was resorted to after a long and unsuccessful course with the ordinary remedies in such cases, and the result was uniform success. He resorts to these injections, which he gives once every four hours, after the subsidence of the acute stage. He is very confident that, properly applied, this solution will effect a cure of the gonorrhœa within from eight to ten days after it has been resorted to.—*Ex.*

**Sure Cure for Corns.**—A. C., who has tried it, is authority for the following: Take one-fourth cup of strong vinegar; crumb finely into it some bread; let it stand half an hour, or until it softens into a good poultice; then apply on retiring at night. In the morning the soreness will be gone, and the corn be picked out. If the corn is a very obstinate one, it may require two or more applications to effect a cure.—*Drug. Circular.*

**Chapped Hands and Frosted Feet.**—At a meeting of the Philadelphia County Medical Society, held November 21, 1883, Dr. Carl Seiler called attention to the value of tincture of benzoin in the treatment of chapped hands and frosted feet. He had used it in a number of cases with much success. It is applied by simply painting it on the skin. The stockings may be prevented from sticking to the feet by rubbing some oil over the benzoin.—*Med. Herald.*

**A New Treatment for Neuralgia.**—The latest agent introduced for the relief of neuralgia is a one per cent. solution of hyperosmic acid, administered by subcutaneous injection. It has been employed in Billroth's clinic in a few cases. One of the patients had been a martyr to sciatica for years, and had tried innumerable remedies, including the application of electricity no fewer than 200 times, whilst for a whole year he had adopted vegetarianism. Billroth injected the above remedy between the tuber ischii and trochanter, and within a day or two the pain was greatly relieved, and eventually quite disappeared. It would be rash to conclude too much from these results, in the face of the intractability of neuralgia to medication, but if it really proves to be as efficacious as considered, hyperosmic acid will be a therapeutic agent of no mean value.—*Lancet.*

**Dr. Willard Parker** is 83 years old; Alonzo Clark, 80; A. C. Post, 77; Isaac E. Taylor and Austin Flint, 71; and Frank H. Hamilton, 70. Some of these distinguished medical men are still in active practice, notwithstanding their great age.—*Cincinnati Lancet and Clinic.*

**Yellow Oxide of Mercury in Ophthalmia.**—Dr. Jonathan Hutchinson says, since he has come to appreciate the value of yellow oxide-of-mercury ointment in the treatment of chronic inflammations of the eye, he has been able to abandon almost entirely the use of blisters, setons, and like painful measures.—*British Medical Journal.*

**Tetanus Cured.**—Drs. Beall & Adams, of Fort Worth, Texas (Tex. Cour. Med.), report a case of tetanus cured. They say: "Knowing the great and rapid fatality of such cases, and believing it futile to employ the remedies usually recommended, but which are powerless to save, we resorted at once to the Hodgen treatment. We gave him eight drops of Fowler's solution and five drops fluid extr. gelsemînum every four hours, for twenty-four or thirty-six hours, and then diminished the former to four or five drops, for some ten or fifteen days, withdrawing the yellow jasmine entirely after the first full effects had been obtained. The patient began to improve in every way, the wound healing kindly, and the improvement in the general condition, and especially with reference to the tetanus condition, progressed *pari passu* with the healing of the wound, and the patient made a good recovery.

**Nevus Treated Successfully by Local Application of Liquor Arsenicalis.**—Mr. W. J. Beatty, L.R.C.P., writes to the British Medical Journal: In my hands it has succeeded admirably, my last eight cases having been cured perfectly and painlessly by the local application of this remedy. The preparation I use is the ordinary liquor arsenicalis of the Pharmacopeia, with which the nevus is to be painted night and morning until ulceration takes place; and I find that the cure is effected in from three to five weeks.—*Louis. Med. News.*

**A Caution About Belladonna Plasters.**—One would hardly suppose that serious results could ensue from the application of a belladonna plaster, yet Dr. Martin J. Fleming reports a case of well-marked belladonna poisoning, relieved by opium treatment, in the Medical Record, January 19, 1884, caused by the application of a plaster to a back that had been somewhat denuded by the use of an irritating liniment. The case suggests the advisability of cautioning patients against applying such plasters over an abraded skin surface.—*Med. and Surg. Reporter.*

**Sierra Salviæ in Remittent Fever.**—We have recently noted that it is generally believed that there are certain cases in which quinine is not admissible. In such, Dr. Francis A. Evans (Nashville Jour. Med. add Surg., December 1883) says that he has had very satisfactory results from the use of sierra salviæ. He uses it in the form of fluid extract, in doses of four drops every two hours, combining it with tincture veratrum or tinct. nux vomica.—*Med. and Surg. Rep.*

**A New Method of Tooth-Drawing.**—A small square of India-rubber, pierced with a central hole, is pushed over the tooth until the upper part of the root is reached. The rubber gradually contracts, pulls on the root, and the tooth is finally enucleated without causing any pain. Four or five days complete the operation, while very slight bleeding and slight swelling of the gum are the only inconveniences. This procedure is recommended by a dentist of Geneva.—*Med. and Surg. Rep.*

